# Complement Activation by Adeno-Associated Virus-Neutralizing Antibody Complexes

**DOI:** 10.1089/hum.2023.018

**Published:** 2023-06-15

**Authors:** Cara West, Joel D. Federspiel, Kara Rogers, Arpana Khatri, Sheila Rao-Dayton, Mireia Fernandez Ocana, Sean Lim, Aaron Michael D'Antona, Sandra Casinghino, Suryanarayan Somanathan

**Affiliations:** ^1^ Pfizer, Inc., Rare Disease Research Unit, Cambridge, Massachusetts, USA; ^2^ Pfizer, Inc., Drug Safety Research & Development, Andover, Massachusetts, USA; ^3^ Pfizer, Inc., Drug Safety Research & Development, Groton, Connecticut, USA; ^4^ Pfizer, Inc., Rare Disease Research Unit, Kit Creek, North Carolina, USA; ^5^ Pfizer, Inc., Biomedicine Design, Cambridge, Massachusetts, USA.

**Keywords:** AAV, complement activation, neutralizing antibodies

## Abstract

Treatment of monogenetic disorders using vectors based on adeno-associated virus (AAV) is an area of intense interest. AAV is non-pathogenic human virus, and preexisting capsid antibodies are prevalent in the population posing a challenge to the safety and efficacy of AAV-mediated gene therapies. In this study, we investigated the risk of AAV-mediated complement activation when sera from a cohort of human donors were exposed to AAV9 capsid. Seropositive donor sera carrying neutralizing antibodies from a previous environmental exposure activated complement when admixed with AAV9 capsids and complement activation was associated with donors who had higher levels of anti-AAV IgG1 antibodies. These findings were consistent with mass spectrometry analysis that identified increased binding of immunoglobulins and complement factors when AAV9 capsids were admixed with seropositive sera. Finally, complement activation was abrogated after IgG-depletion using affinity columns or serum pretreatment with an IgG degrading enzyme. Overall, these results demonstrate an important role of preexisting neutralizing antibodies in activating complement; a risk that can be mitigated by using adequate immunosuppression strategies when dosing seropositive patients with vector.

## INTRODUCTION

Adeno-associated virus (AAV)-mediated gene therapy has been approved to treat retinal blindness, neuromuscular disorders, and dyslipidemia.^1–3^ The clinical success with AAV gene therapy has led to several ongoing late-stage clinical trials to treat a myriad of rare monogenetic disorders.^4^ An impediment to successful AAV gene transfer is the presence of preexisting antibodies from wild-type AAV infection in patients.^5–8^ Antibodies thwart *in vivo* gene transfer, and transgene expression has been ineffectual after vector administration to seropositive non-human primates (NHPs) and human subjects.^9,10^ Consequently, exclusion of seropositive patients with preexisting antibodies above a neutralizing level (titer cutoff) has been implemented in several clinical trials. These measures have provided benefit by avoiding the complexities that accompany vector administration to seropositive patients. Nevertheless, there is interest in extending AAV gene therapies to additional patients by raising the titer cutoff.

These proposals have been bolstered by reports of stable FIX expression in a gene therapy trial that inadvertently administered AAV5.FIX vector to seropositive patients.^11^ While no adverse events have been reported in clinical trials that dosed seropositive patients, vector administration to patients with preexisting antibodies does carry safety risks. Antigen–antibody (AAV-Ab) immune complexes can trigger complement activation, enhance phagocytosis, activate a cytokine storm, or induce antibody-dependent cellular cytotoxicity.^12^ AAV vectors were also demonstrated to activate antigen-presenting cells and activate complement when exposed to donors with high titer antibodies.^13^ Consequently, there is a need to improve our understanding of host–capsid interactions to determine possible outcomes upon vector administration to seropositive patients.

In this study, we pursued a strategy to investigate AAV9 complement activation following capsid interaction with seropositive and seronegative human serum. Immunoglobulins and complement factors bound capsid at higher levels when exposed to seropositive serum resulting in complement activation that could be reduced by immunoglobulin (IgG/IgM) removal or enzymatic IgG depletion.

## MATERIALS AND METHODS

### AAV vectors

The empty AAV9 vector used in mass spectrometry and complement activation assays was purified from cell lysates following triple transfection of HEK293 cells (Pfizer, Rare Diseases Research Unit). Clarified cell lysates were run on an AAV9 affinity column and the eluate was polished using anion exchange chromatography. The peaks corresponding to the empty capsids were collected, buffer exchanged, and resuspended in phosphate-buffered saline (PBS) containing 5% sorbitol at a titer of 1.3 × 10^14^ vp/mL based on protein content. The AAV9 vectors expressing firefly luciferase driven from a chicken-beta actin 6 promoter (AAV9.CB6.PI.ffluciferase) were obtained from the Viral Vector Core at UMass (MA, USA), and the AAV9.CMV.lacZ vector used in the binding antibody (BAb) assays was purchased from Vigene Biosciences (MD, USA). All vectors were endotoxin free and the vectors containing transgene were correct by restriction digest.

### Donor serum

Serum was obtained from healthy human adult donors through the Pfizer Research Support Programs in Cambridge, MA, USA, and Groton, CT, USA, in accordance with the IRB guideline. Donor serum was heat inactivated at 56°C for 30 min before use in neutralizing antibody (NAb) and BAb assays.

### NAb assay

A transduction inhibition assay was used as a surrogate to evaluate the presence of neutralizing antibodies.^14^ Serum was serially diluted using serum-free Dulbecco's modified Eagle medium (DMEM) twofold from a starting dilution of 1:5 (up to 1/40). Diluted serum samples were incubated for 60 min with an equal volume of AAV9.CB6.PI.ffluciferase vector (UMASS vector core) in serum-free DMEM. Stock vectors had a titer of 3 × 10^13^ vg/mL, endotoxin free, correct inverted terminal repeat integrity, and resuspended in PBS containing 5% glycerol. HEK293T cells were seeded overnight (18–24 h) at a density of 60,000 cells/well in a 96-well plate. After 18–24 h, wild-type human adenovirus 5 was added at a multiplicity of infection (MOI) of 100 (Cat. No. VR-1516; ATCC) for 2 h. Media were aspirated and replaced by vector–serum complexes at an MOI of 1 × 10^4^ vg/cell and incubated for 2 h.

At the end of incubation, 10% heat-inactivated fetal bovine serum was added to the wells. Cells were lysed and assayed for luciferase transgene expression the next day (18–24 h) using the Bright-Glo™ Luciferase Assay System (Promega Corporation, Madison, WI, USA) as per the manufacturer's recommendation. Plates were read on Perkin Elmer Envision plate reader and samples were considered NAb positive when serum diluted at least fivefold and admixed with AAV9 vectors resulted in >50% reduction in transgene expression compared with vectors incubated without serum. Average relative light units (RLUs) of wells with vector in the absence of neutralizing serum were 5,110 ± 1,063 RLUs, which was several fold higher than control wells (30 ± 10 RLUs) that received no vector.

### BAb assay

A plate-based assay was used for detecting antibodies that bound vector.^14^ Briefly, 96-well ELISA plates (Corning, USA) were first coated overnight with 2 × 10^9^ vg/well of AAV9.CMV.lacZ vector (titer: 1.13 × 10^13^ vg/mL; Vigene Biosciences, MD, USA) diluted in PBS pH 7.5. The following day plates were washed three times with TBS-tween (tris-buffered saline with 0.05% tween 20) and blocked for 1 h with 5% nonfat dry milk (Cat. No. 190915; Andwin Scientific). Plates were then washed three times with TBS-tween before adding 100 μL of serum, diluted twofold in PBS, starting from an initial dilution of 1:5, to the plate.

After incubation, plates were washed and bound antibodies were detected using the following reagents, anti-human IgG1 HRP (goat anti-human, Cat. No. 62-8420; Invitrogen), anti-human IgG2-HRP (mouse anti-human, Cat. No. MH-1722; Thermo Fisher Scientific), anti-human IgG3-HRP (mouse anti-human, Cat. No. MH-1732; Thermo Fisher Scientific), anti-human IgG4 (mouse anti-human, Cat. No. MH-1742; Thermo Fisher Scientific), and anti-human IgM (mouse anti-human, clone HP6083, Cat. No. 05-4920; Thermo Fisher Scientific). Samples were considered positive when the signal in wells incubated with sample was greater than threefold the levels observed in control wells incubated without serum.

### Complement activation assay

Serum from human donors was thawed in a 37°C water bath and immediately placed on ice before incubation with 2 × 10^12^ empty AAV9 capsids for 1 h at 37°C. After incubation, samples were returned to ice. Complement fragment, C3a, was measured using the Quidel MicroVue Complement C3a Plus kit (Quidel Corporation, San Diego, CA, USA) according to the manufacturer's protocol.

### Antibody depletions

Pierce™ Protein A/G magnetic agarose beads (Thermo Fisher Scientific, Waltham, MA, USA) were prepared according to the manufacturer's protocol. To deplete IgG, beads were incubated with serum from human donors, previously shown to be positive for AAV9-induced complement activation. To obtain IgG/IgM-depleted serum, the IgG-depleted serum was then incubated with anti-human IgM−agarose antibody (Sigma, St. Louis, MO, USA), and then centrifuged. AAV9 (empty capsid)-induced complement activation was assessed as described above.

### Gene Ontology analysis

Gene Ontology (GO) analysis was done using Pantherdb.org to perform a GO Biological Process enrichment against a human serum proteome background file. REVIGO was used to summarize the GO findings and generate tree maps for visualization. Principal component analysis (PCA) was done using ClustVis. Volcano plot illustrations were made in Instant Clue. Differential protein analysis was performed comparing serum samples from donors with an *in vitro* complement response to AAV9 capsids with those samples that did not; proteins with a false discovery rate (FDR) of 0.05 or less were considered differential.

### Data sharing statement

The mass spectrometry (Supplementary Methods) proteomic data have been deposited to the MassIVE resource and can be downloaded at ftp://massive.ucsd.edu/MSV000091454/

### IgG degrading enzyme from *Streptococcus pyogenes* degradation

Serum samples from human donors, previously shown to be positive for AAV9-induced complement activation, were incubated with 20 μg/mL of IgG degrading enzyme from *Streptococcus pyogenes* (IdeS) for 15 min at 37°C. Following IdeS incubation, AAV9 capsid-induced complement activation was assessed as described earlier. IgG depletion in human sera was verified by immunoblotting IgG.

## RESULTS

### Identification of AAV9 seropositive donors with binding and neutralizing antibodies

**Figure 1.**
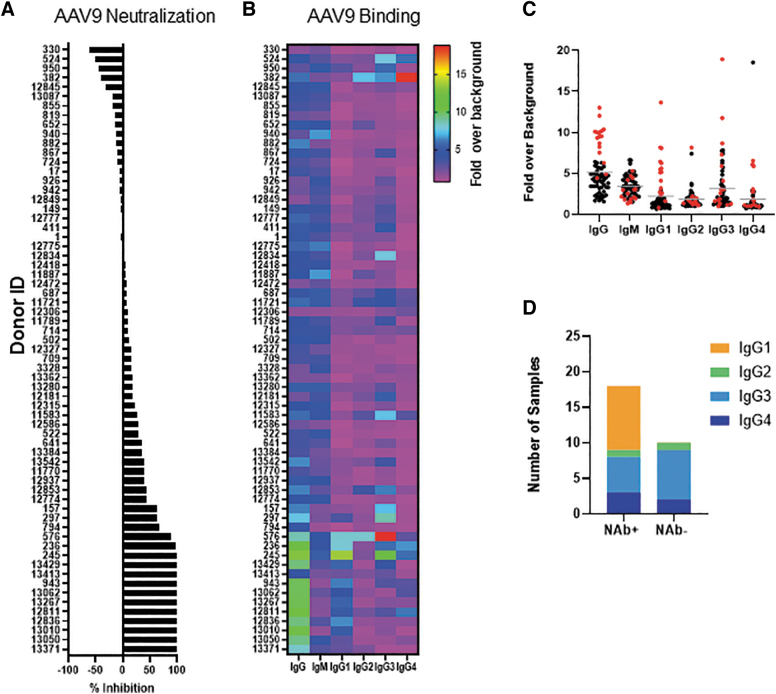
Anti-AAV9 binding and neutralizing activity in human donors. **(A)** Percent AAV9 transduction inhibition when admixed with human donor sera. *Horizontal plot* lists donor sera *top* to *bottom* in increasing order of transduction inhibition. Sera that enhanced transduction (negative inhibition) are displayed at the *top*. **(B)** Heat map showing IgG and IgM antibody binding intensities as a color gradient among subjects. **(C)** Violin plot of the mean and breadth of the BAb response among NAb+ (*red dots*) or NAb− (*black dots*) subjects. **(D)** Antibody binding among NAb+ and NAb− subjects stratified by IgG subclasses. AAV9, adeno-associated virus 9; BAb, binding antibody; NAb+, NAb seropositive; NAb−, NAb seronegative.

We executed a series of studies to evaluate AAV9 seropositivity in a cohort of healthy human subjects (*N* = 65) using two different assays. Donors were mostly (85%) Caucasian adults (age 26–61) and of mixed gender (29 females and 36 males). First, a transduction inhibition assay was used as a surrogate for establishing AAV9 NAb seropositivity. Sera from a proportion (16/65; 25%) of subjects neutralized an AAV9 vector expressing firefly luciferase and inhibited transduction >50%; the inhibition threshold for seropositivity (Fig. 1A). Inhibition levels among seropositive subjects varied and serum from 12/16 NAb subjects completely inhibited transduction (98–100%) when admixed with vector, whereas serum from four other subjects (157, 297, 794, and 576) inhibited transduction at lower levels (63–88%). Forty-nine subjects were NAb seronegative (NAb−) and transduction inhibition was <50%. Interestingly, sera from 11/49 subjects enhanced vector transduction and is reported as negative % transduction inhibition (−10% to −60%).

Next, we compared seropositivity by the BAb assay. An ELISA-based BAb assay was used to isotype and subtype antibodies that bound vector (Fig. 1B). Isotyping revealed a clear association between NAb seropositivity and IgG BAbs with 14/16 NAb seropositive (NAb+) subjects also having high IgG BAbs (>sixfold over baseline). This contrasted with only 3/49 NAb− subjects having high IgG BAbs. Subjects 794 and 13,413 did not have significantly higher BAbs despite being NAb+, suggesting that the presence of nonimmunoglobulin (non-Ig) serum neutralizing factors may have affected vector transduction.

The strong association between IgG and NAb seropositivity led us to investigate IgG subclasses that bound vector. The four IgG subclasses (IgG1, IgG2, IgG3, and IgG4) have different structures and functions although sharing 90% amino acid identity.^15^ IgG subclass profile revealed that BAbs were mostly IgG1, IgG3, and IgG4 (Fig. 1C). Among the subclasses, the association of IgG1 BAbs with seropositive subjects was higher. On average, IgG1 levels were >5.5 ± 3-fold over baseline among NAb+ subjects and <1.3 ± 0.4-fold among NAb− subjects. While BAbs were also detected in several sera that did not neutralize vector transduction, there was a notable lack of increased IgG1 subclass antibodies (Fig. 1D). IgM levels in this study were not associated with serum neutralizing activity and sera from three subjects (940, 12,775, and 11,887) with the highest IgM BAb levels did not neutralize vector.

### Complement activation by AAV seropositive sera

**Figure 2.**
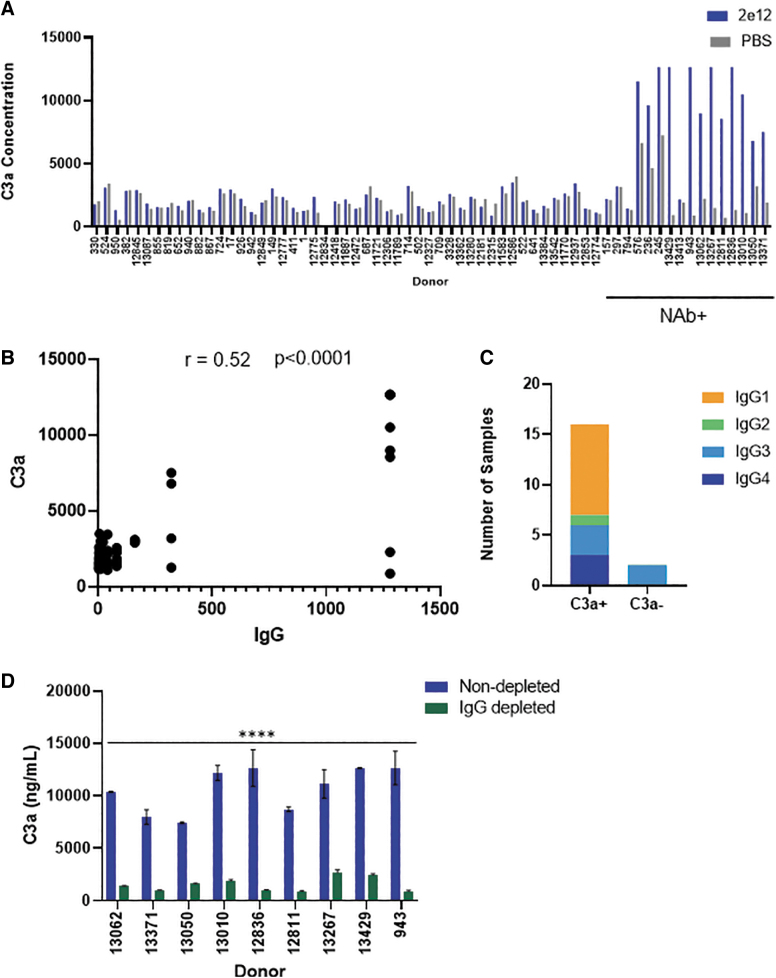
Complement activation in NAb+ human donor serum. **(A)** Complement activation was evaluated by quantifying C3a generation when individual donor sera were incubated with 2 × 10^12^ vg of AAV9 vector (*blue bars*) or with PBS (*gray bars*; negative control). Seropositive sera that neutralized vector transduction in the NAb assay are indicated below the plot. **(B)** Correlation between C3a levels and IgG binding titers among all donors. Nonparametric Spearman correlation coefficient (*r*) was computed between IgG titers and C3a activation levels. **(C)** Distribution of donors with the different anti-AAV IgG subclasses among those who did (C3a+) or did not (C3a−) activate complement. **(D)** Complement activation after immunoglobulin depletion. Nondepleted (*blue bars*) or antibody-depleted (*green bars*) donor sera were incubated with AAV vectors before evaluating complement activation. Significant difference in C3a generation after antibody depletion is represented by *p < 0.05.

We next evaluated *in vitro* complement activation by incubating AAV9 capsid with all 65 sera. Complement activation can occur via the classical, alternate, and lectin pathways. All three pathways converge and result in generation of C3a, C5a, and the membrane attack complex. Increased C3a generation has been used as a “pathway agnostic” indicator of complement activation. We measured C3a generation as a surrogate for complement activation after complexing vector with serum from all 65 subjects (Fig. 2A). Complement activation led to higher C3a generation when AAV9 capsid was admixed with serum from 12/65 subjects. We next evaluated the correlation between subjects with higher titers of antibodies with complement activation.

There was moderate correlation (Spearman correlation *r* = 0.52) between subjects with high IgG binding antibodies and C3a levels (Fig. 2B). While all 12 complement activating sera were also NAb+, it was difficult to establish a correlation with NAbs due to the greater variability in NAb titers and lack of sufficient donors. Interestingly, four donors (157, 297, 794, and 13,413) did not activate complement despite their seropositive status. Lack of complement activation by two of the four subjects (794 and 13,413) is consistent with their nonantibody-mediated vector neutralization noted earlier. However, the other two subjects (157 and 297) had IgG3 binding antibodies, yet did not activate complement.

While these findings may suggest that the IgG3 subclass is less efficient at activating complement upon binding AAV9 capsids, the dearth of subjects with IgG3 binding antibodies does not support a broad conclusion. Binning of complement activating sera by IgG subclasses also revealed a preponderance of subjects with anti-AAV IgG1 subclass antibody (Fig. 2C) and is consistent with the known abundance and proinflammatory effector functions of this subtype.^15^ Complement activation was absent when vectors were incubated with any NAb− serum. Overall, these results indicate a strong association between AAV vector neutralization and complement activation by subjects with higher levels of (>10 × of baseline) preexisting anti-AAV IgG antibodies.

### Classical pathway of complement activation

AAV complement activation by NAb+/IgG+ subjects implicates activation via the classical pathway involving AAV-Ab complex formation. To confirm classical pathway activation, we investigated C3a generation after complexing AAV9 capsid with sera devoid of IgG/IgM antibodies. Complement activating sera from nine seropositive subjects were selected for the IgG/IgM depletion studies. C3a levels were 10,644 ± 2,118 ng/mL when capsids were incubated with seropositive sera. In contrast, complement activation was reduced and C3a levels were 1,529 ± 676 ng/mL upon vector incubation with Ig-depleted serum (Fig. 2D). As a note, C3a levels in healthy subjects range from 20 to 156 ng/mL^16^ and levels can increase several fold during viral infections.^17^ However, C3a levels even at baseline and following AAV vector incubation with serum were significantly higher than the reported *in vivo* levels.

The higher C3a levels observed in our *in vitro* studies are consistent with a previous report that noted a similarly higher C3a activation levels upon adenovirus incubation with human plasma. C3a levels in this study was several fold higher when compared to *in vivo* C3a levels after virus administration.^18^ The disconnect between the high C3a levels observed *in vitro* and *in vivo* may arise due to *in vivo* regulation of C3a levels. Immunoglobulin depletion had no effect on C3a generation in the absence of AAV confirming the specificity of AAV-mediated complement activation (Supplementary Fig. S1).

### Direct binding of complement factors to AAV

Complement activation by seropositive sera led us to investigate serum factors that bound vector using mass spectrometry (Supplementary Fig. S2). We first evaluated factors that associated with vector when complexed with sera from 31 seropositive and seronegative subjects (14 NAb^+^/C3a^+^ and 17 NAb^−^/C3a^−^). As a note, 11/17 NAb^−^/C3a^−^ donors came from screening a second cohort of donors (data not shown) as low sample volume from several cohort 1 donors prohibited their inclusion in these studies. Vector incubation with all 31 sera identified several factors that bound capsid (Supplementary Table S1).

To identify serum factors that only bound capsid in the presence of antibodies, we used differential analysis of capsid-bound proteins. Capsids were incubated either with antibody-replete or antibody-depleted sera. Factors that bound capsid under the two conditions were then compared to identify those that require AAV-Ab recognition as a prerequisite for binding. IgG/IgM antibodies were depleted from 27/31 donor sera (14 NAb+ and 13 NAb−). Insufficient serum from four donors led to their exclusion from these depletion studies. Depletion resulted in >98% reduction in total IgGs and >90% reduction in IgM levels (Supplementary Figs. S4 and S5, respectively). Forty-two factors bound vector following IgG/IgM depletion irrespective of the donor serostatus (Supplementary Table S2).

**Figure 3.**
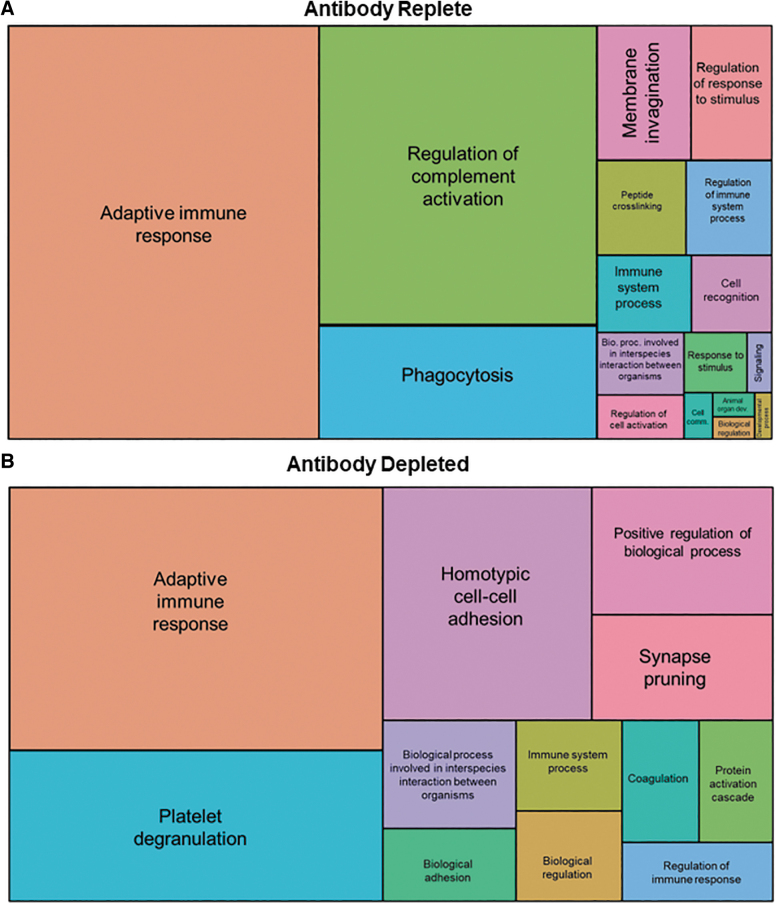
GO biological process enrichment analysis. Mass spectrometry analysis of factors that associated with AAV9 when incubated with antibody-replete **(A)** or antibody-depleted **(B)** serum. Boxes represent vector-associated factors involved in indicated pathways and are sized by the adjusted *p*-value of the enrichment against a human serum proteome. GO, Gene Ontology.

Factors that bound capsid were then classified based on their involvement in biological and cellular processes. Most of the bound factors were immunoglobulins, complement proteins, and proteins involved with adaptive immunity as assessed by GO enrichment (Fig. 3A). Antibody depletion led to a remarkable reduction in the binding of complement-related proteins (Fig. 3B). In other changes, there was increased binding of factors involved in phagocytosis in antibody-replete serum that was notably absent after antibody depletion. Instead, there was increased binding of factors involved in cellular adhesion and platelet degranulation after antibody depletion. Increased binding of these factors may have resulted from recognizing capsid moieties that were previously inaccessible due to competition from antibodies.

**Figure 4.**
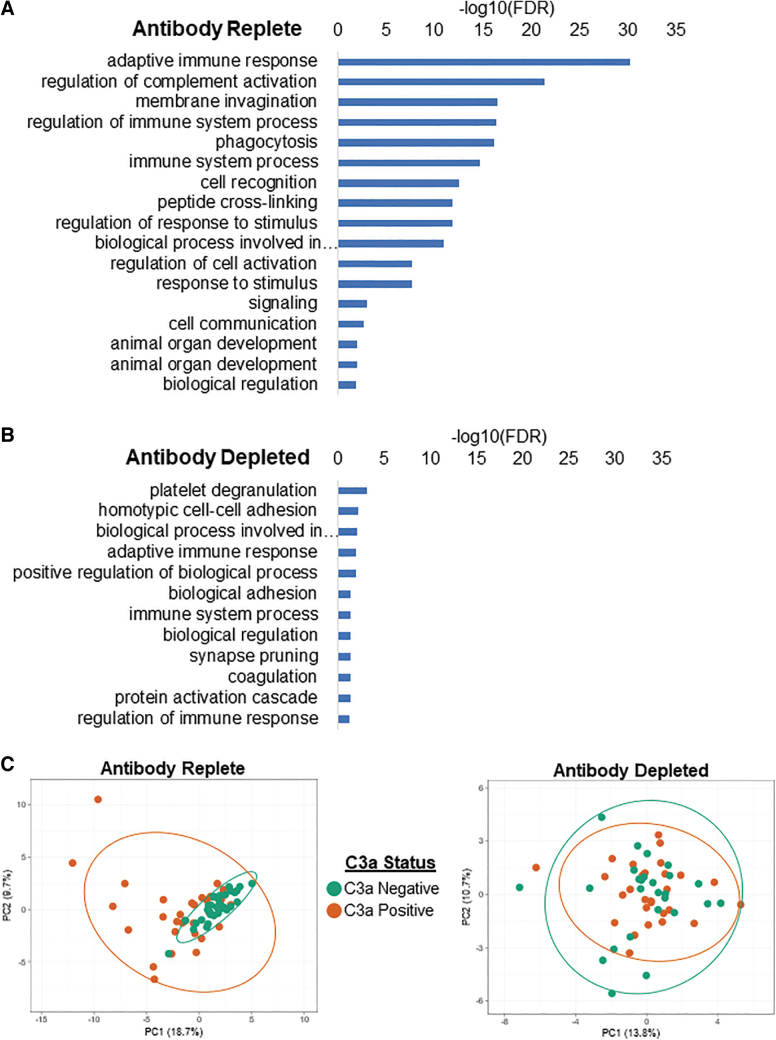
Differential association of factors in antibody-replete and antibody-depleted serum with AAV. FDR of factors involved in various cellular pathways that associated with capsid before **(A)** and after **(B)** antibody depletion. **(C)** PCA of antibody-replete and antibody-depleted serum. Each *dot* on the plot represents an individual subject serum sample that either activated (*red*) or did not activate (*green*) complement. FDR, false discovery rate; PCA, principal component analysis.

As a note, adaptive immune response GO terms were still enriched following antibody depletion, however, the degree of enrichment was greatly reduced from an FDR (−log_10_FDR) of 30.2 in the antibody-replete samples to a −log_10_ FDR of 1.98 in the antibody-depleted samples (Fig. 4A, B). PCA of binding factors was performed to differentiate complement activating and nonactivating subjects. At the level of the whole interactome, there was tight clustering of C3a-negative, and a more disperse clustering of C3a-positive, subjects when analyzing their antibody-replete sera (Fig. 4C). However, no clustering of subjects was observed following antibody depletion.

The previous analysis yielded data on 42 factors enriched in the presence of antibodies irrespective of serostatus (Supplementary Table S2). To identify the subset of factors that only bound capsid in the presence of NAbs, we compared bound factors among seropositive and seronegative subjects (Supplementary Fig. S3). This differential analysis identified 22 factors enriched only in seropositive subjects who also activated complement (Table 1). Most factors were either immunoglobulins (13/22) or complement proteins (6/22). A notable exception was lacritin, a glycoprotein more commonly found in human tears, that was most associated with vector upon incubation with seropositive sera.

**Table 1.** Differential adeno-associated virus 9 capsid-bound proteins (false discovery rate ≤0.05) in complement activating (C3a positive) versus nonactivating (C3a negative) sera

Accession	Description	Gene Symbol	Log_2_ Fold Change (C3a+/C3a−)	*q*-Value (C3a+/C3a−)
Q9GZZ8	Extracellular glycoprotein lacritin	LACRT	4.69	3.47E-06
A0A0A0MS15	Immunoglobulin heavy variable 3–49	IGHV3-49	1.84	1.99E-02
P0C0L4	Complement C4-A	C4A	1.72	2.18E-02
P00736	Complement C1r subcomponent	C1R	1.44	8.72E-03
P0DOY3	Immunoglobulin lambda constant 3	IGLC3	1.33	1.33E-02
A0A0A0MT89	Immunoglobulin kappa joining 1	IGKJ1	1.28	1.11E-02
A0A0C4DH36	Probable nonfunctional immunoglobulin heavy variable 3–38	IGHV3-38	1.27	1.99E-02
P02745	Complement C1q subcomponent subunit A	C1QA	1.13	7.78E-03
A0A0C4DH38	Immunoglobulin heavy variable 5–51	IGHV5-51	1.11	3.94E-03
P02747	Complement C1q subcomponent subunit C	C1QC	1.03	1.36E-02
P0DOY2	Immunoglobulin lambda constant 2	IGLC2	0.95	1.35E-02
P01834	Immunoglobulin kappa constant	IGKC	0.86	3.61E-03
P01860	Immunoglobulin heavy constant gamma 3	IGHG3	0.73	2.87E-02
P0C0L5	Complement C4-B	C4B	0.73	3.22E-02
P01857	Immunoglobulin heavy constant gamma 1	IGHG1	0.71	3.61E-03
P02671	Fibrinogen alpha chain	FGA	0.69	1.33E-02
P01619	Immunoglobulin kappa variable 3–20	IGKV3-20	0.63	1.20E-02
P02746	Complement C1q subcomponent subunit B	C1QB	0.60	7.43E-03
P01591	Immunoglobulin J chain	IGJ	0.55	4.55E-02
B9A064	Immunoglobulin lambda-like polypeptide 5	IGLL5	0.51	2.03E-02
P01876	Immunoglobulin heavy constant alpha 1	IGHA1	0.39	1.09E-02
P62937	Peptidyl-prolyl cis-trans isomerase A	PPIA	0.27	1.35E-02

**Figure 5.**
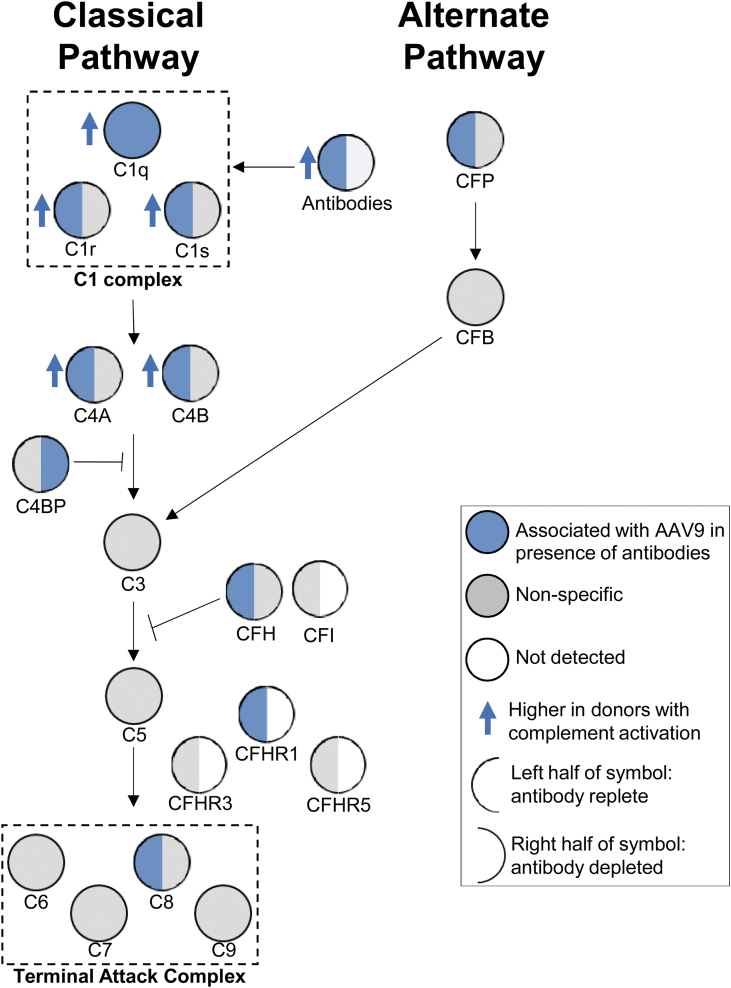
Classical complement pathway components associate with AAV9 in donors with complement response. Simplified complement pathway showing association of upstream classical pathway complement proteins with vector. For a given complement factor, the *left semicircle* represents vector association upon incubation with antibody-replete sera, while the *right half* represents vector association after antibody depletion. *Circles* and *semicircles* are colored based on: (1) factor bound vector (*blue*), (2) bound nonspecifically (*gray*), or did not associate with vector (*white*). The members of the C1 complex and terminal attack complex are *boxed*. *Blue arrows* pointing up indicate factors that bound vector at significantly higher levels among subjects who activated complement.

Among the complement factors, there was strong association of factors that are part of the classical pathway of complement activation (Fig. 5). As an example, factors that make up the C1 complex and are part of the classical pathway (C1r and C1s) bound AAV9 in the presence (left blue semicircle) or absence (right gray semicircle) of antibodies. However, these factors associated at significantly higher levels upon vector incubation in the presence of complement activating sera (blue arrow), suggesting increased recruitment in the presence of AAV-specific antibodies. While C1r and C1s could directly bind capsid in the absence of antibodies, C1q required the presence of antibodies (blue circle). Similar analysis did not identify increased alternate pathway components complement factor P and complement factor B binding capsids. Factors downstream of C3 in the activation pathway were also not enriched in binding capsid. Overall, these findings lead us to infer that interaction of C1q components with capsid in the presence of neutralizing/binding antibodies was required for initiation of complement activation observed in our studies.

### Complement activation after IdeS treatment

While the risk of rampant complement activation can be reduced by using the anti-C5 monoclonal antibody, an alternate approach is to reduce the overall burden of anticapsid antibodies. The immunoglobulin G-degrading enzyme of Streptococcus pyogenes (IdeS) specifically cleaves human IgG into F(ab′)_2_ and Fc fragments and has been approved as a pretreatment to reduce IgGs in patients undergoing human leukocyte antigen-incompatible kidney transplantation.^19^ IdeS preadministration led to successful AAV transduction of NAb+ NHPs.^20^ As complement activation correlated with NAb presence, we hypothesized that IdeS pretreatment would eliminate or reduce complement activation. Complement activation was evaluated from eight NAb+ sera with or without IdeS pretreatment. IdeS pretreatment completely cleaved IgG into F(ab′)_2_ and Fc fragments (data not shown).

**Figure 6.**
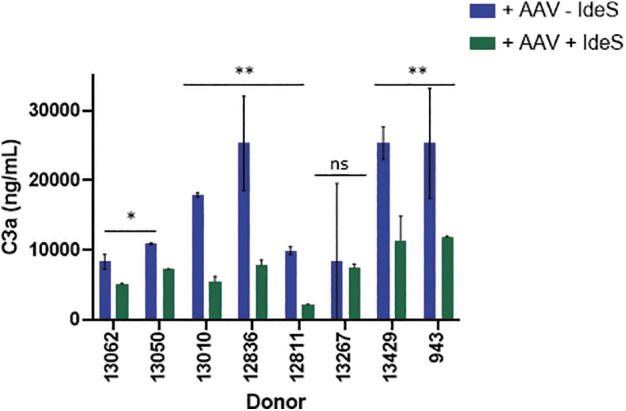
IdeS pretreatment abrogates complement activation by neutralizing sera. Sera from eight donors with neutralizing antibodies were evaluated for AAV9 capsid-mediated complement activation with or without IdeS pretreatment. IdeS-treated (*green bars*) or untreated (*blue bars*) serum samples were incubated with vector before evaluating complement activation by measuring C3a concentrations. Donors with significant differences in C3a following IdeS treatment are represented by *p < 0.05. IdeS, IgG degrading enzyme from *Streptococcus pyogenes*; ns, nonsignificant.

As expected, sera from all eight seropositive subjects demonstrated complement activation and C3a generation when complexed with vector (Fig. 6), although the levels of activation varied among subjects. IdeS pretreatment significantly reduced C3a generation by 58% ± 20% when compared with untreated samples in all, but one subject.

## DISCUSSION

Positive serostatus is often an exclusion criterion in AAV clinical trials that require intravenous vector infusion.^21^ The basis for seropositive patient exclusion was based on preclinical studies and clinical trials demonstrating inefficient gene transfer following systemic vector administration to antibody-positive subjects. AAV8.GFP vector administration to nonhuman primates was associated with reduced liver gene transfer in animals with NAb titers >1:5.^9^ Similar NAb titers (>1:5) also abrogated vector transduction in macaques administered AAV8.FIX or AAV5.FVIII-SQ vectors.^22,23^ In the clinic, AAV2.FIX vector administration to NAb+ patients was associated with reduced transgene expression. Peak FIX expression in two subjects who received the highest dose of AAV2.FIX vector (2E12 vg/kg), and had preexisting NAb titers of 1:2 and 1:17, was 11% and 3%, respectively.^10^

In a second AAV.FIX-R338L trial that used a hyperfunctional transgene, FIX activity was lower in one patient with an NAb titer of 1:1 when compared with nine others who had titers <1:1.^24^ These findings have led to implementation of clinical protocols that have excluded seropositive patients. Seropositivity is commonly investigated using two assays; a cell-based transduction inhibition assay that quantifies anticapsid neutralizing antibodies (TI or NAb assay) or a biochemical assay that measures total capsid binding antibodies (TAb or BAb assay).^14^ These assays aim to identify and exclude seropositive patients with preexisting antibodies. However, seronegative patients dosed with vector may not be completely devoid of anti-AAV antibodies. Assay sensitivity prohibits accurate antibody detection below a predefined level.^25^

Thus, thresholds may reflect the limit below which transduction is not sufficiently impacted by preexisting immunity. This increases the possibility that some patients administered AAV gene therapy may have low levels of preexisting humoral immunity or seropositive patients may still be included if antibodies do not prevent vector transduction.^21^ Nevertheless, preexisting antibodies carry the risk of activating a type II hypersensitive reaction.

In this study, we investigated this risk in a cohort of AAV9 seropositive subjects by evaluating complement activation by capsid–antibody complexes. First, with a few exceptions, there was high correlation between the presence of AAV9 NAbs and BAbs. While most NAb+ sera also had high levels of IgG BAbs, differential analysis revealed significantly lower binding of IgG subtypes. For instance, some donor sera had 10-fold higher IgG BAbs, yet a <2-fold increase in IgG subclass binding. It is possible that total IgG detection, agnostic to the subclass, detects binding of all antibodies that increases the overall signal intensity when compared with detection of any single subclass. However, there was also evidence for higher detection of some IgG subclasses compared with total IgG in the same subject. It is important to note that the assay and reagents used to detect IgG or IgG subclasses are different and could explain these discrepancies.

Next, vector incubation with seropositive serum resulted in increased association between complement factors and immunoglobulins that resulted in complement activation. Analysis of factors found associated with vector was consistent with the classical pathway of complement activation mediated by increased binding of complement C1q subcomponents.^26^ C1q subcomponents have previously been reported to bind AAV9 when incubated with seronegative serum.^27,28^ Our findings confirm these earlier observations, but also demonstrate increased binding of these complement components upon exposure to seropositive sera. Complement components are known to bind AAV capsids that increase their uptake and activation of macrophages.^29^ It is conceivable that the recognition and binding of capsids by antibodies result in increased recruitment of complement factors that activates a signaling cascade. Besides complement components, human serum albumin (HSA) and galectin-3 binding protein (G3BP) are known to bind AAV vectors enhancing and inhibiting transduction, respectively.^28,30^

While we did observe G3BP bound AAV9 vector in our studies consistent with the earlier observations, we did not detect HSA binding. An interesting finding of vector incubation with seropositive sera was the association of the tear glycoprotein lacritin. While lacritin is found in human tears where it acts as an inducer of autophagy to restore health, it is also a human plasma protein.^31^ The reason for lacritin association with AAV is unclear. Lacritin does bind syndecan-1, a heparin sulfate proteoglycan found on antigen-presenting cells and T cells.^32^ It is possible that binding of AAV9 capsids by lacritin in seropositive serum targets its uptake into immune cells that activates a more potent immune response. However, it is unclear how lacritin binds vector in the presence of antibodies and further studies would be required to establish the nexus between capsid, antibodies, immune cells, and lacritin in enhancing vector immunogenicity.

It should be noted that an unexpected finding from our studies was the enhanced transduction in the presence of seronegative sera from some subjects. It is possible that enhancement was mediated by non-Ig factors similar to the previous reports of HSA-mediated transduction enhancement. Such enhancement may be lower in the presence of neutralizing antibodies.

Not all seropositive subject sera activated complement and serum from two donors (794, 13,413) neutralized vector, yet did not activate complement. These donors also had the lowest levels of BAbs when compared with other donors who neutralized vector. It is conceivable that transduction inhibition by these donor sera was most likely mediated by non-Ig neutralizing factors. A previous study has reported similar non-Ig-mediated neutralization of AAV5 vectors in a screen of healthy human subjects.^33^ Non-Ig factors that inhibit transduction are less likely to activate complement based on the well-established pathways of complement activation. Our BAb assay was limited to the detection of IgG and IgM isotypes, and vector neutralization/complement activation by other isotypes cannot be ruled out. IgG and IgM are canonically the isotypes most associated with a strong complement activation.^34^

However, based on our finding, seropositive subjects with higher anti-AAV9 IgG levels, especially those with higher IgG1 levels, were more likely at risk of complement activation if dosed with an AAV9 vector.

Interestingly, two subjects (157 and 297) with NAbs and BAbs did not activate complement. It is unclear why these donors did not activate complement although the subclass of IgG present in their sera (IgG3) is known to activate complement.^34^ The dose of vector used in our complement activation assays may have been insufficient to trigger IgG3-mediated activation. On the contrary, IgG3 antibodies may be less likely to trigger complement activation upon binding AAV9 vector or require synergistic interaction with other pathway factors to mediate complement activation that were absent in our assay.

Clinical trials that administered high-dose AAV9 vector to patients with Duchenne muscular dystrophy have reported complement activation.^35,36^ An interesting question is why complement activation occurred when dosing seronegative patients? The severe adverse events involving thrombotic microangiopathy and atypical hemolytic uremic syndrome associated with complement activation occurred within 2 weeks postvector dosing.^37^ It is possible that the delayed response results from complement activation due to *de novo* antibodies that increase after a primary humoral immune response to capsid antigens. This presupposes that capsid antigens remain in circulation long enough for binding and activation by antibodies. AAV9 pharmacokinetic studies do suggest slower capsid blood clearance that may enhance immunogenicity by the formation of AAV-antibody complexes.^38,39^

While there are limitations to our study, the main drawback is the use of an *in vitro* assay to measure complement activation. Nevertheless, these findings raise the possibility that dosing seropositive patients increases the risk of type I hypersensitivity reactions leading to complement activation that requires further investigation. It is intriguing why complement activation was not observed in some clinical trials that did dose seropositive patients with AAV vectors.^10,11^ The antibody levels in these patients may have been insufficient to trigger the complement cascade. It is impossible to compare titers across AAV clinical trials due to a lack of standardization and differences in the conduct of the assay.^40^ Alternatively, we cannot discard the possibility that the dose of vector used in these trials may have been below the threshold required to induce complement activation. Another pertinent difference is the vector used in these trials.

Our studies presented here, and the Duchenne muscular dystrophy clinical trials, used an AAV9 vector and the response to other capsids may differ based on factors binding capsids, host immune responses, and capsid pharmacokinetics.^38^

Immunosuppression regimens are often used to de-risk activation of host immunity following AAV vector administration. Our studies suggest that the use of an effective immunoglobulin depletion strategy may enhance the safety of AAV gene therapy by reducing the risk of complement activation. The enzymatic approach used in this study does not deplete IgM, a potent activator of complement. Preexisting IgM antibodies may be less of a concern in patients who may have been previously infected with wild-type AAV since IgM responses to common human parvoviruses peak within days and are undetectable after 17 weeks.^41^

In conclusion, the requirement to broadly transduce multiple tissues currently requires systemic high-dose AAV vector administration.^42^ The assays used here, and other similar assays, can be used to prescreen seropositive patients to identify those at a higher risk of complement activation when intravenously administering high-dose AAV vectors.

## Supplementary Material

Supplemental data

Supplemental data

Supplemental data

Supplemental data

Supplemental data

Supplemental data

Supplemental data

Supplemental data
