# Supplementary material for: Complement Activation by Adeno-Associated Virus-Neutralizing Antibody Complexes
Source: Hum Gene Ther. 2023 Jun 15;34(11-12):554–66. doi: 10.1089/hum.2023.018 (PMC10282828; doi:10.1089/hum.2023.018)
Supplement: Supplemental data [file Supp_Method.docx]

*Biotinylated anti-AAV9 antibody*

The anti-AAV9 antibody was generated in-house and biotinylated as follows. 200ug of antibody was reacted with 10mM EZ-Link Sulfo-NHS-LC-Biotin (Thermo Fisher, A39257) at a 50:1 biotin:antibody ratio for 90 minutes in the dark at room temperature. Labeling was quenched by the addition of 2% of the total reaction volume of 2M Tris-HCl. The labeled antibody was desalted using a 7K MWCO Zeba Spin column (Thermo Fisher, 89882) according to the manufacturer’s instructions.

*Mass Spectrometry* analysis

Empty AAV9 particles (1x10^12^ vp) were first admixed with 50µL of donor serum. The AAV-serum complexes were either immediately subject to immunoprecipitation (IP) or incubated at 37ºC for an hour before proceeding with IP. In parallel studies, serum samples were first depleted of IgG and IgM before complexing with AAV9 particles. Each donor was analyzed with a matched negative control IP for every condition which lacked any AAV9 capsid but was otherwise the same in all respects. To these samples, 450µL of PBST, 1X final concentration of protease and phosphatase inhibitor (HALT, Fisher Scientific, 78444), 3µg of biotinylated anti-AAV9 antibody, and 10µL of pre-washed magnetic streptavidin C1 beads (Fisher Scientific, 65002) were added. This mixture was then incubated at 4ºC for 30 min with continuous rotation. Bound AAV9 capsids were captured magnetically and washed 4X with PBST and 1X with PBS either manually or through use of an automated liquid handling platform (Hamilton Microlab Star). Capsids and associated bound proteins were eluted via low pH, then reduced with 5mM dithiothreitol (Fisher Scientific, USA), and alkylated with 10mM iodoacetamide (Fisher Scientific, USA). Samples were then digested either by adjusting the elution to 5% SDS and using S-Trap micro columns (ProtiFi, C02-micro-80) according to the manufacturer’s instructions using the High Recovery Protocol or via in-solution digest. Samples digested in-solution were heated at 100°C for 8 min to denature the capsid and then cooled to room temperature prior to addition of trypsin. For both digestion methods, digested peptides were desalted using C18 columns (NEST Group Inc, USA) and elutions were dried down in a vacuum concentrator. Peptides were resuspended in 10µL and 20% was used for LC-MS/MS analysis.

Peptides were analyzed by LC-MS/MS using an Ultimate 3000 UPLC (Thermo Scientific) coupled online to an EASYSpray ion source and an Exploris 480 mass spectrometer (Thermo Scientific). Peptides were separated on an EASYSpray C18 column (75 μm × 50 cm) heated to 50°C using mobile phases A (0.1% formic acid in water) and B (0.1% formic acid in 90% acetonitrile) at a flow rate of 250 nL/min over 60min with a linear gradient from 3%B to 35%B. Peptides were ionized at 1.7 kV and an MS1 survey scan was conducted from 350-1600 *m/z*, with an automatic gain control (AGC) target of 3e^6^ at 120,000 resolution. Data-dependent scans on the top 15 precursors were performed with an isolation window of 1.2 *m/z*, a normalized collision energy of 30%, an AGC target of 1x10^5^, and a maximum injection time of 100 ms at 30,000 resolution.

Data were searched using Proteome Discoverer (PD) 2.4 (Thermo Scientific) with the Byonic (Protein Metrics) node against a human reference FASTA file appended with the AAV9 VP1 sequence and common contaminants (20,409 total sequences, downloaded 1/3/2020). Protein identifications were filtered to a false discovery rate (FDR) of 1% at the peptide and protein level and label-free MS1 quantitation was performed. Data were exported from PD and analyzed further in Excel and Prism 9.0 (GraphPad). Data from different sample sets were combined by scaling each set to the other sets. This was accomplished by calculating the mean abundance of a protein detected in each dataset and then calculating the mean of the datasets means. Then the mean from each dataset was divided by the mean of means to generate a scaling factor for each dataset. Every sample in a given dataset was then scaled by this scaling factor on a protein-by-protein basis in order to combine the data. Proteins that specifically associate with the AAV9 capsid were identified by filtering as follows: within each group (complement response or no response at 4°C or 37°C) an average fold change of MS1 abundance for each protein in log_2_ space was calculated versus the matched controls and a multiple comparison adjusted t-test (2-step FDR calculation) was performed in Prism. Proteins with an average log2 fold change of bait/control of at least 2 and with a multiple comparison adjusted p-value of 0.01 or less which also had at least as many spectral counts in the bait IPs as the control IPs were considered specific.
