# Supplementary figures and images for: Complement Activation by Adeno-Associated Virus-Neutralizing Antibody Complexes
Source: Hum Gene Ther. 2023 Jun 15;34(11-12):554–66. doi: 10.1089/hum.2023.018 (PMC10282828; doi:10.1089/hum.2023.018)

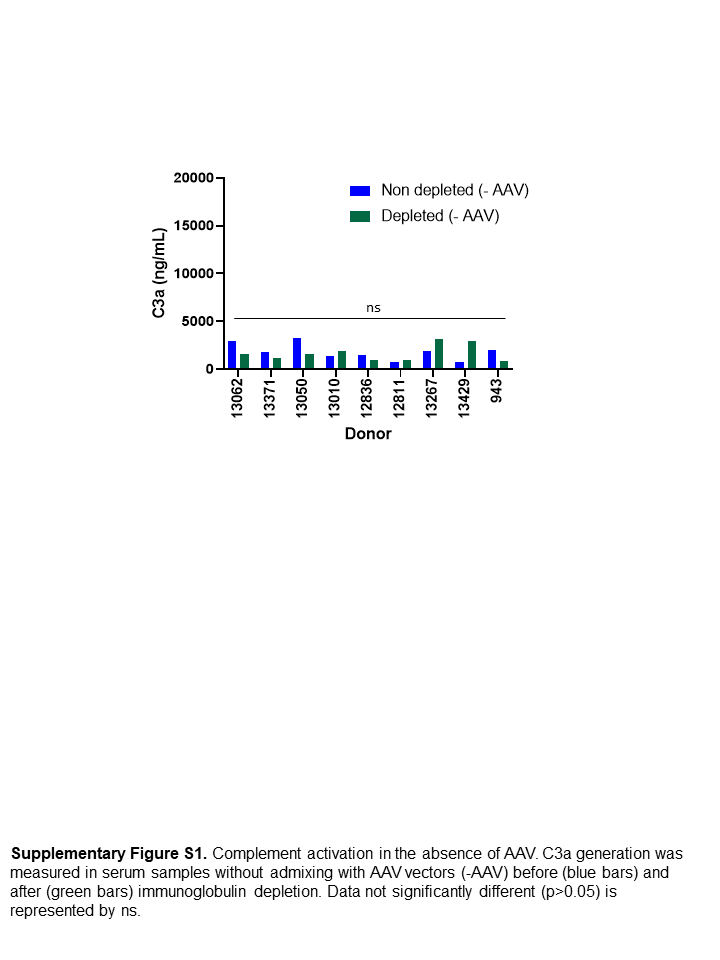

Supplement: Supplemental data [file Supp_figS1.docx]

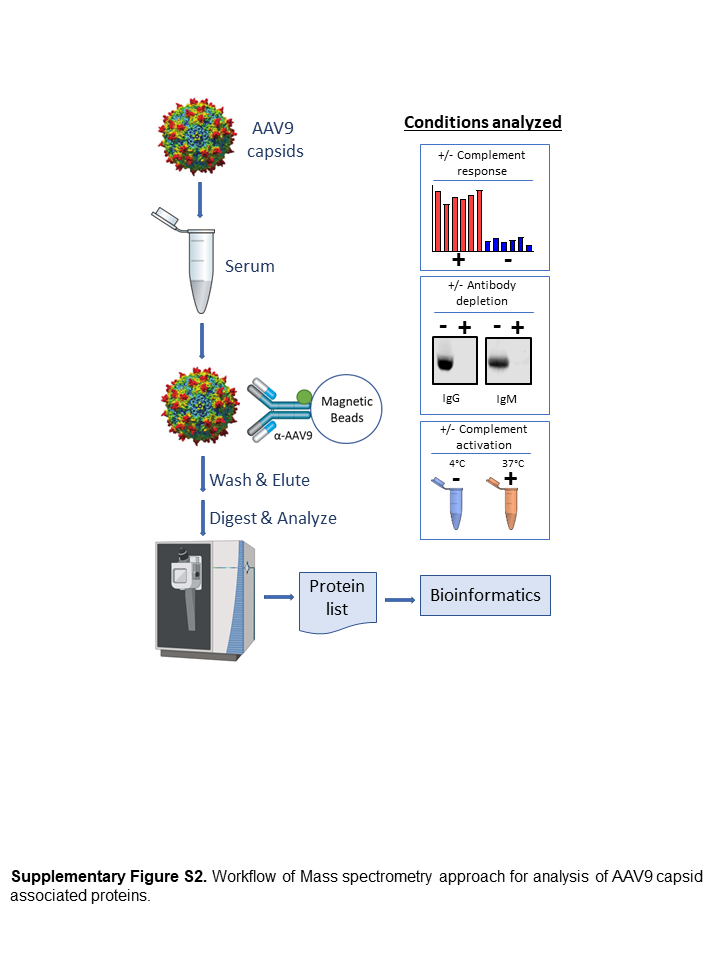

Supplement: Supplemental data [file Supp_figS2.docx]

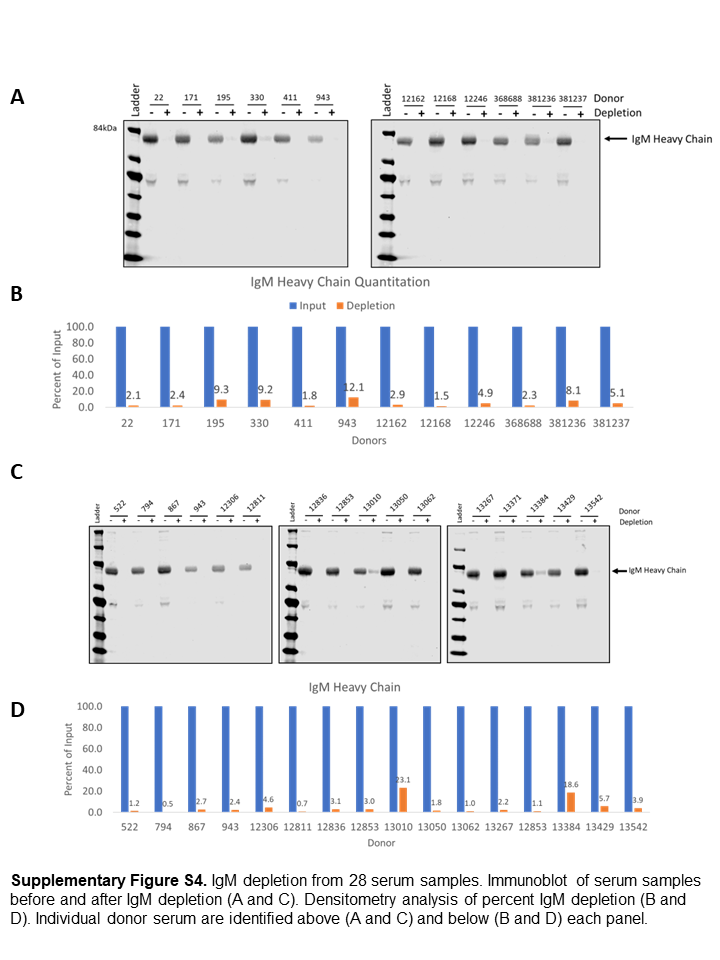

Supplement: Supplemental data [file Supp_figS4.docx]

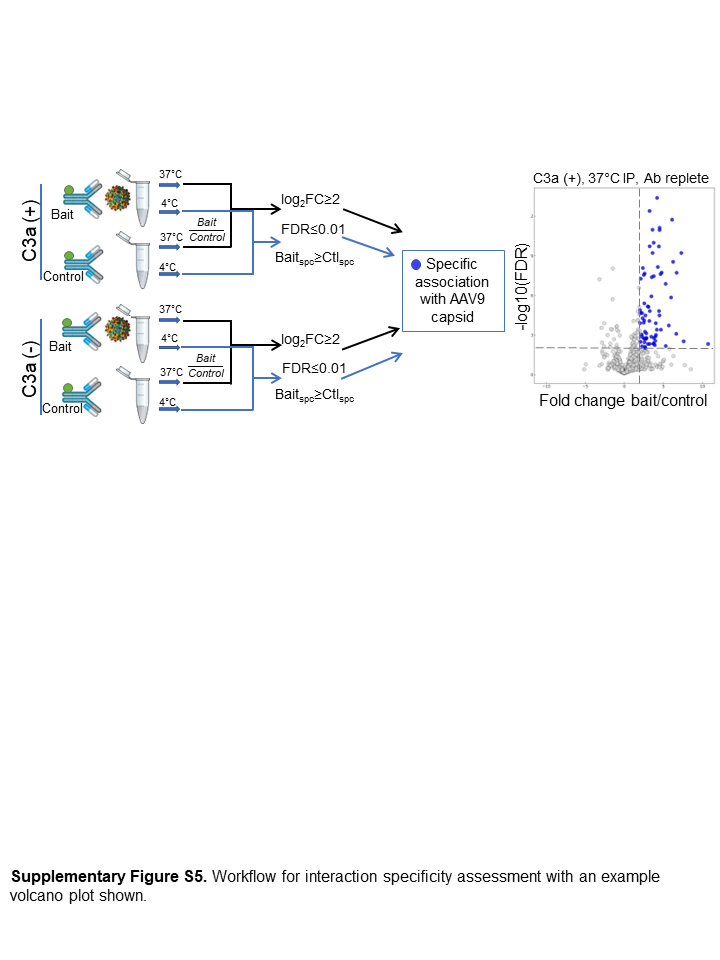

Supplement: Supplemental data [file Supp_figS5.docx]

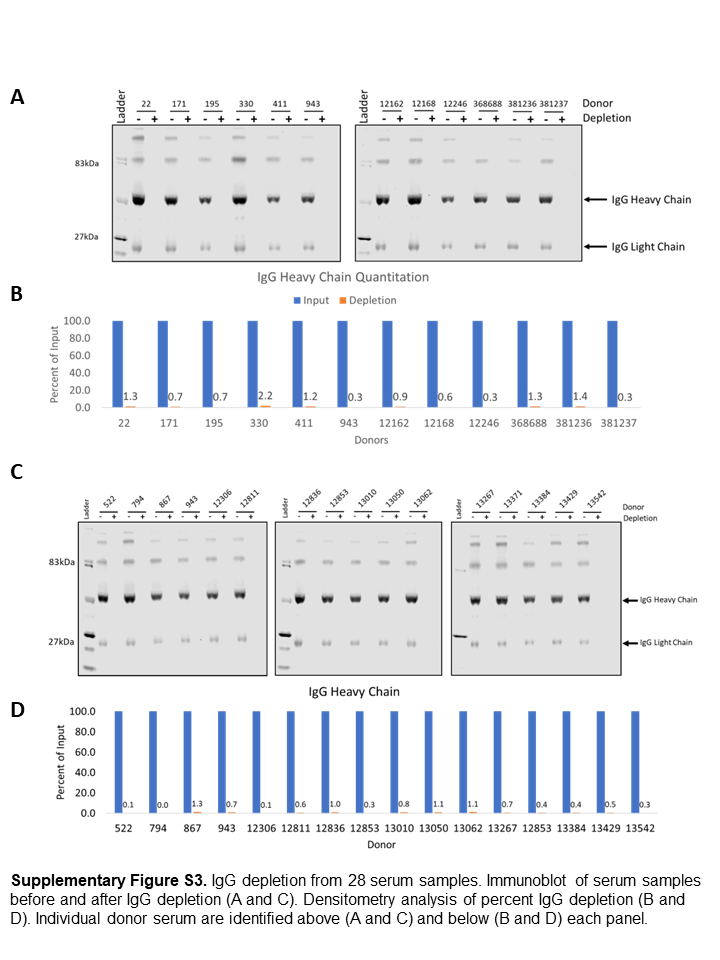

Supplement: Supplemental data [file Supp_figS3.docx]
